# A new species of *Peckoltia* (Siluriformes, Loricariidae) from the rapids of the Rio Tocantins‐Araguaia basin, Brazil

**DOI:** 10.1111/jfb.70235

**Published:** 2025-10-04

**Authors:** Felipe Arian Andrade de Araújo, Marlon Felipe Chumber Ferreira, Aline Nascimento Silva, Wolmar Benjamin Wosiacki

**Affiliations:** ^1^ Postgraduate Program in Biodiversity and Evolution Museu Paraense Emílio Goeldi Belém Brazil; ^2^ Department of Zoology, Ichthyology Museu Paraense Emílio Goeldi Belém Brazil

**Keywords:** Ancistrini, Hypostominae, rheophilic species, suckermouth armoured catfish

## Abstract

Here, we describe a new species of *Peckoltia* (Loricariidae; Hypostominae) from the rapids of the Rio Tocantins‐Araguaia basin, previously identified as *Peckoltia vittata*, using an integrative taxonomy approach. The new species is distinguished from congeners by ventral region presenting diffuse stripes on surface, not presenting spots or blotches on head and body, elongated odontodes on cheeks reaching the pectoral‐fin spine when adpressed to body, space between the eyes not entirely covered by a blotch, parieto‐supraoccipital moderately elevated, not forming an apparent crest, 14–26 teeth on premaxilla, 15–24 teeth on dentary, diminute plates with short odontodes on base of pectoral fins, and anterior part of urogenital opening, a less developed suspensorium, a prominent lateral wall of metapterygoid channel with sturdy base, including conspicuous ornated edges, adductor palatine crest forming a diminute roughly perceptible salient, diminute hyomandibula concave triangular shape. The phylogeny based on Cytb molecular marker recovered the new species placed with congener species, congruent with the morphological classification findings.

## 1 INTRODUCTION

As a member of Hypostominae (Loricariidae), *Peckoltia* is classified in the recently designated subtribe Peckoltini (Armbruster & Lujan, 2024). This group is one of the most speciose fish groups for the Neotropics, presenting impressive morphological diversity (Armbruster et al., 2018; Fricke et al., 2025; Lujan et al., 2015). The genus is considered monophyletic based on molecular evidence (Lujan et al., 2017; Lujan & Conway, 2015), but taxonomically, it still lacks a proper diagnosis, even though, generically, it can be characterized by specimens presenting saddles on body and dentary teeth row <90° (Armbruster et al., 2018; Armbruster & Lujan, 2016; Lujan et al., 2015). This armored catfish group is broadly distributed across the Amazon River basin, with 22 described species (Armbruster et al., 2018, Fricke et al., 2025).

Revisions of specimens deposited in museums previously identified as *Peckoltia vittata* (Steindachner 1881) increased the knowledge of the diversity within the genus. This species is likely distributed in the range recognized for the genus, occurring in tributaries of the Amazon River basin and across all the Upper Río Orinoco (Armbruster & Lujan, 2016). Armbruster (2008) had hypothesized that specimens morphologically similar to *P. vittata* could represent putative undescribed species. Lately, this assumption was corroborated with the description of *Peckoltia compta* Oliveira, Zuanon, Rapp Py‐Daniel, Rocha 2010, *Peckoltia greedoi* (Armbruster et al., 2015), *Peckoltia lujani* (Armbruster et al., 2015) and *Peckoltia wernekei* (Armbruster & Lujan, 2016), all previously identified as *P. vittata*.

Here, we describe a new species of *Peckoltia* Pleco from the Rio Tocantins‐Araguaia basin. Likewise, a phylogenetic analysis is included to confirm its relationship with other *Peckoltia* species.

## 2 METHODS

### 2.1 Ethics statement

The care and use of experimental animals complied with Brazilian animal welfare laws, guidelines and policies as approved by under licensing 70940–1 SISBIO/IBAMA.

### 2.2 Taxonomic description

Morphometric measurements were made with direct corporal measurements taken with digital callipers to the nearest 0.1 mm. Counts and measurements follow Armbruster (2003). Partial measurements of standard length (SL) are expressed as a proportion of SL, and partial measurements of head length (HL) are expressed as a proportion of HL. Institutional abbreviations are listed in Sabaj (2020). Comparative data were retrieved based on original *Peckoltia* species descriptions (Armbruster, 2003, 2008; Armbruster et al., 2015; Armbruster & Lujan, 2016; Armbruster & Werneke, 2005; Ribeiro et al., 2010) and from specimens deposited at MPEG. The complete morphometric dataset is presented in Data S1.

For morphological analysis, we conducted X‐ray scanning and three‐dimensional image reconstructions X‐ray scanning with nanocomputerized tomography of the described species and *P. vittata*. Samples were scanned using a GE v|tome|x m dual tube 300/180 kv system.

Each specimen was scanned with an energy beam of 80 kV and a flux of 80× μA using a 360° rotation and then reconstructed into the 4096 × 4096 matrix of 1536 slices. The final computed tomography (CT) reconstructed images were exported with a minimum resolution of 6.099 μm. The images were segmented manually using 3DSlicer (Fedorov et al., 2012) to isolate the suspensorium. The scale bars of holotype and comparative material are presented in millimetres.

### 2.3 DNA extraction, amplification and sequencing

Genomic DNA was extracted from a muscle tissue sample of the paratype specimen (D157, MPEG 039494) using the DNeasy Blood and Tissue kit (Qiagen, Hilden, Germany) according to the manufacturer's protocol. To assess the extracted DNA quality, samples were stained with GelRed and electrophoresed on a 1% agarose gel for 40 min at 60 V. Amplification of Cytb fragment followed thermocycler conditions according to Lujan et al. (2015). Each polymerase chain reaction (PCR) had a final volume of 15 μL, containing 2.5 μL of dNTPs (1.25 mM), 1.5 μL of 10× buffer, 0.6 μL of MgCl_2_ (1.5 mM), 0.6 μL of each primer (50 ng/μL), approximately 100 ng (1 μL) of total DNA, 0.1 μL of Taq DNA polymerase (5 U/μL) and purified water to complete the volume. Positive PCR products were purified using the ExoSAP‐IT PCR Product Cleanup Reagent (Thermo Fisher Scientific) according to the manufacturer's protocol and subsequently sequenced using the BigDye kit (ABI Prism Terminator Cycle Sequencing Ready Reaction‐PE Applied Biosystems) on an ABI 3500 automatic capillary sequencer (Applied Biosystems).

### 2.4 Molecular analysis

Sequences were edited and aligned in Geneious version 8.1.7. For phylogenetic reconstruction two methods were applied: maximum likelihood (ML) and Bayesian inference (BI) analyses. The BI analysis was performed using MrBayes software version 3.2.7 (Ronquist et al., 2012) with the GTR + G evolutionary model and partition schemes estimated using PartitionFinder2 (Lanfear et al., 2016), with Spectracanthicini species as out‐group. Two independent runs were performed with four Markovian chains (MC3) based on 10 million generations, with tree sampling at every 10,000 generations, discarding 25% as burn‐in. The ML tree was inferred using raxmlGUI 2.0 (Edler et al., 2021), with 1,000 bootstrap replicates adopting the GTR + G as evolutionary model.

## 3 RESULTS

### 3.1 *Peckoltia amjikin*, new species

**FIGURE 1:**
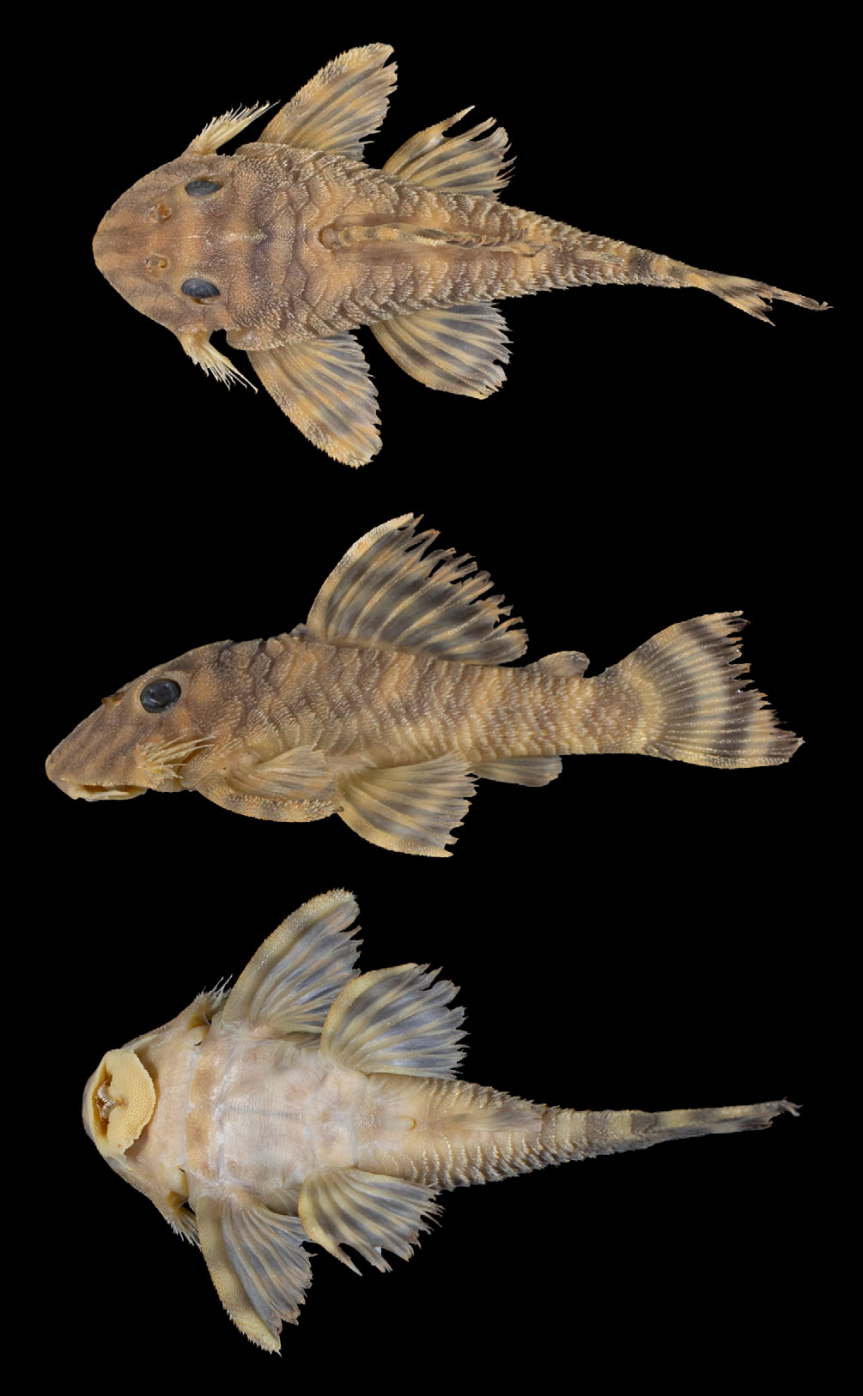
Holotype of *Peckoltia amjikin* (MPEG 40735), 64.8 mm SL, Brazil, Tocantins, Bom Jesus do Tocantins, Rio Tocantins basin.

Zoobank accession number: urn:lsid:zoobank.org:pub:C997C7EA‐95D0‐411A‐B414‐3CED8183DAC3 (Figure 1; Table 1).

**TABLE 1:** Morphometric data of *Peckoltia amjikin* from the Rio Tocantins basin.

	*N*	H	Range	Mean	SD
SL (1–20)	18	64.8	51.8–64.8	58.7	4.1
SL%					
Pre‐dorsal length (1–10)	18	29.3	41.8–52.0	45.4	2.4
Head L. (1–7)	18	23.9	36.2–40.8	37.7	1.3
Head dorsal L. (7–10)	18	5.4	5.9–10.7	7.9	1.5
Cleithral W. (8–9)	18	22.4	30.4–36.5	34.3	1.5
Head‐pectoral L. (1–12)	18	19.1	27.0–32.8	30.1	1.5
Thorax L. (12–13)	18	13.9	21.0–27.6	23.7	1.7
Pectoral‐spine L. (12–29)	18	18.6	28.7–35.3	31.3	1.7
Abdominal L. (13–14)	18	16.3	22.2–26.2	24.4	1.1
Pelvic‐spine L. (13–30)	17	16.5	25.0–28.8	26.8	1.1
Postanal L. (14–15)	18	20.4	28.6–35.7	31.5	1.7
Anal‐fin spine L. (14–31)	18	10.7	14.2–18.4	16	1.1
Dorsal‐pectoral D. (10–12)	18	18.9	27.6–34.0	30	1.7
Dorsal spine L. (10–11)	17	17.3	24.9–36.6	28.6	3.0
Dorsal‐pelvic D. (10–13)	18	17.2	20.4–31.0	26.5	2.6
Dorsal‐fin base L. (10–16)	18	16.5	19.6–27.4	24.8	1.9
Dorsal‐adipose D. (16–17)	18	7.8	11.4–20.7	14.1	2.1
Adipose‐spine L. (17–18)	18	7.6	9.0–12.7	11	1.0
Adipose‐spine caudal D. (17–19)	18	11.0	13.5–19.6	17	1.6
Caudal peduncle Dp. (15–19)	18	7.7	10.8–13.0	11.8	0.6
Adipose‐low caudal D. (15–17)	18	16.1	19.6–25.9	24.1	1.6
Adipose‐anal D. (14–17)	18	12.2	15.2–22.7	18.4	1.7
Dorsal‐anal D. (14–16)	18	11.1	15.6–18.9	17.3	0.8
Pelvic‐dorsal D. (13–16)	18	19.3	23.9–3.9	28.5	2
HL%					
Head‐eye L. (5–7)	18	8.5	31.4–42.1	36.1	2.5
Orbit diameter (4–5)	18	4.9	18.5–24.4	21.5	1.7
Snout L. (1–4)	18	14.2	47.7–70.3	58.4	4.8
Internares W. (2–3)	18	3.2	10.7–16.8	13.3	1.8
Interorbital W. (5–6)	18	10.1	11.7–50.0	39.0	7.8
Head Dp. (7–12)	18	16.4	60.1–81.2	71.1	5.3
Mouth L. (1–24)	17	11.4	32.5–56.4	45.1	6.5
Mouth W. (21–22)	17	10.7	39.1–55.6	46.2	5.4
Barbel L. (22–23)	18	4.6	10.3–24.8	17.4	3.7
Dentary tooth cup L. (25–26)	18	3.4	6.2–16.8	11.9	2.7
Premaxillary tooth cup L. (27–28)	18	2.5	8.2–12.4	10.4	1.3

*Note*: Morphometric data, related to body, except standard length, are given as percentages of the standard length (SL%); morphometric data of the head are expressed as percentages of head length (HL%). Numbers in parentheses refer to the landmarks in Armbruster (2003).

Abbreviations: D, distance; Dp, depth; H, holotype values; HL, head length; *N*, number of individuals; SD, standard deviation; SL, standard length; W, width.

#### 3.1.1 Holotype

MPEG 40735, 64.8 mm SL, Brazil, Pará State, Bom Jesus do Tocantins, Rio Tocantins, 5°20′11.9″ S 48°51′15.5″ W, 7 November 2019.

#### 3.1.2 Paratypes

MPEG 039494, 1, 54.9 mm SL, Brazil, Pará State, São João do Araguaia, Rio Tocantins: Ilha das Cabras, 5°17′37.0″ S 48°57′32.7″ W, 6 November 2019. MPEG 039541, 5, 52.9–64.1 mm SL, Brazil, Pará State, Bom Jesus do Tocantins, Rio Tocantins: beach in front of Jaú Island, 5°20′11.9″ S 48°51′15.5″ W, 7 November 2019. MPEG 039604, 3, 54.3–63.6 mm SL, Brazil, Pará State, Bom Jesus do Tocantins, Rio Tocantins: Cajú Amigo, almost in the front to the São João do Araguaia city, 5°20′27.0″ S 48°47′18.3″ W, 9 November 2019. MPEG 039321, 1, 59.0 mm SL, Brazil, Pará State, São Geraldo do Araguaia, Rio Araguaia: Sobradinho, 6°09′48.0″ S 48°25′29.0″ W, 30 July 2022. MPEG 039302, 2, 59.4–59.8 mm SL, Brazil, Tocantins State, Xambioá, Rio Araguaia: Remanso do Boto, 6°22′48.0″ S 48°23′16.0″ W, 28 July 2022.

#### 3.1.3 Non‐types

MPEG 039165, 5, 54.0–31.0 mm SL, Brazil, Pará State, São Geraldo do Araguaia, Rio Araguaia: Paredão das Três bocas, 6°08′53.0″ S 48°22′56.0″ W, 30 July 2022. MPEG 039337, 2, Brazil, Maranhão State, Porto Franco, Rio Tocantins: Pedral da Ceval, próximo a Cachoeira seca, 6°26′11.0″ S 47°25′56.0″ W, 2 August 2022. MPEG 039374, 3, Brazil, Tocantins State, Tocantinópolis, Rio Tocantins: Pedral da Santa, 6°19′20.0″ S 47°24′18.0″ W, 2 August 2022.

#### 3.1.4 Diagnosis

**FIGURE 2:**
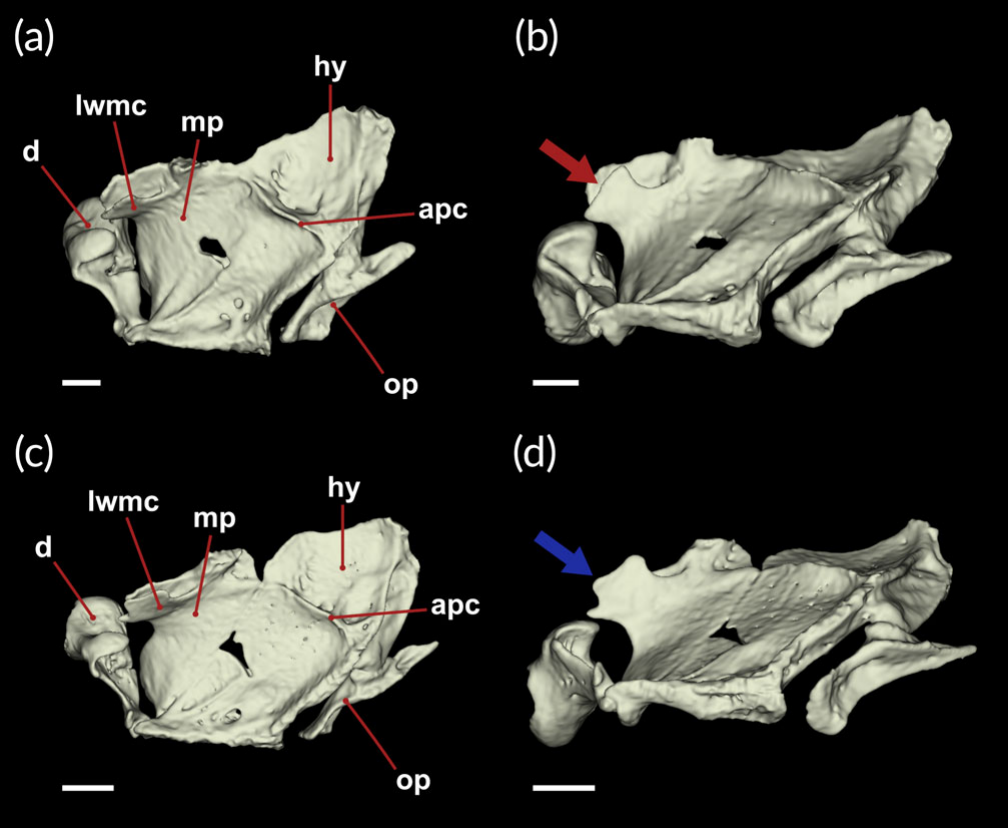
Three‐dimensional computed tomography renderings of the suspensorium in dorsal view. (a) Holotype of *Peckoltia amjikin* (MPEG 40735); (b) lateral view of the suspensorium highlighting the lateral wall of the metapterygoid channel with sinuous edges; (c) suspensorium *of Peckoltia vittata* (MPEG 13428) in dorsal view; (d) lateral view of the suspensorium of *P. vittata* highlighting the lateral wall of the metapterygoid channel and its ornamentation. apc, adductor palatini crest; d, dentary plate; hy, hyomandibula; lwmc, lateral wall of metapterygoid channel; mp, metapterygoid; op, opercle. Scale bar = 1 mm.

*Peckoltia amjikin* is distinguished from congeners, except *P. compta*, *P. greedoi*, *Peckoltia multispinis* (Holly 1929), *P. vittata*, *Peckoltia vermiculata* (Steindachner 1908) and *P. wernekei*, by not presenting spots or blotches on head and body (vs. with dots or blotches with a variety of size on head, body and fins). Differ from *P. multispinis* by lower lips with rounded papillae (vs. branched fimbriate papillae). Can further be distinguished by *P. vittata* by elongated odontodes on cheeks reaching the pectoral‐fin spine when adpressed to body (vs. adults lacks odontodes on cheeks), space between the eyes not entirely covered by a blotch (vs. dark blotch between eyes and on snout), parieto‐supraoccipital moderately elevated not forming an apparent crest (vs. parieto‐supraoccipital with rounded crest), by diminute plates with short odontodes on base of pectoral fins and anterior part of urogenital opening (vs. abdomen plated from throat to urogenital opening); prominent lateral wall of metapterygoid channel with sturdy base, including sinuous edges (vs. lateral wall of metapterygoid channel base and edges same size and shape, presenting ornaments); adductor palatine crest forming a diminute, almost perceptible salient (vs. adductor palatine crest forming a slightly salient; Figure 2a). Differ from *P. wernekei* by upper and lower jaws with <25 teeth (vs. teeth frequently 26 or more). Further can be separated by *P. greedoi* by seven saddles along the body (vs. three saddles). Differ from *P. compta* by four oblique bars on body behind head, reaching further midventral plate rows series without fading (vs. five or more well‐marked bars reaching below median plate series), further by dark bars crossing membranes of dorsal fin (vs. dark spots restricted to fin rays). *Peckoltia amjikin* differs from *P. vermiculata* by yellow stripes of the head obliquely positioned on head, not larger than eye orbit (vs. lines originating on parieto‐supraoccipital).

#### 3.1.5 Description

Morphometric data are presented in Table 1. Largest analysed specimen 64.8 mm SL. Body short, robust and deep. Greatest body depth at dorsal‐fin origin. Body widest at pectoral‐fin insertion, narrowest at end of caudal peduncle. Snout not completely rounded. Caudal peduncle markedly laterally compressed. Ventral profile straight from snout to caudal‐fin origin. Dorsal profile convex from tip of snout to dorsal‐fin origin, declining in straight line from dorsal‐fin spine to dorsal procurrent caudal‐fin ray. Supraoccipital inconspicuous. Interorbital space flat, slightly elevated on eye edges. Parieto‐supraoccipital uniformly elevated medially.

**FIGURE 3:**
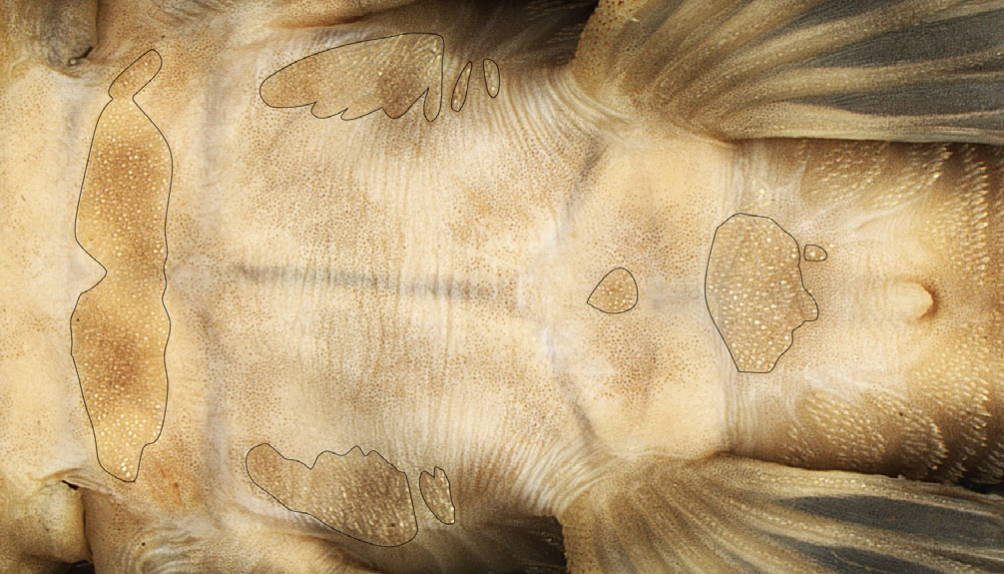
Ventral view of *Peckoltia amjikin* illustrating the diminute plates positioned in the pectoral girdle on lateral the side of abdomen and anteriorly to urogenital opening.

Body plated dorsal and lateral of head, trunk and caudal peduncle. Diminute plates supporting odontodes on pectoral girdle, on lateral of abdomen and anteriorly to urogenital opening (Figure 3).

Head not completely rounded in dorsal view. Eyes moderately sized with iris operculum. Hypertrophied odontodes 14–30 (27*, mode 30) on evertible cheek plates; largest odontodes almost reaching completely base of pectoral fin. Ventral surface of head without plates. Oval area at extremity of snout naked, without plates.

Dorsal‐fin rays, II,7; spine and branched rays supporting small odontodes. Pectoral‐fin rays, I,6, pectoral spine supporting small odontodes on dorsal, anterior and ventral surfaces, larger than those on branched rays and more developed on tip of spine; pectoral‐fin tips reaching less than half of pelvic‐fin spine when adpressed, with locking mechanism. Adipose‐fin spine with small odontodes. Pelvic‐fin rays, i,5.

Anal‐fin rays, i,4; bearing odontodes. Caudal‐fin rays, i,14,i; emarginate, ventral lobe longer than dorsal; caudal‐fin spines and branched rays bearing odontodes similar in size.

Body plates not carenate. Diminute odontodes along body. Pair of pre‐dorsal plates between supraoccipital and origin of dorsal fin. Median plates 23 or 24 (23*, mode 23); mid‐dorsal plates 23*–24 (mode 23); mid‐ventral plates 22–24 (23*, mode 23); five rows of plates on caudal peduncle. Row of mid‐ventral plates folded above pectoral girdle, continuous ridge to cleithrum.

**FIGURE 4:**
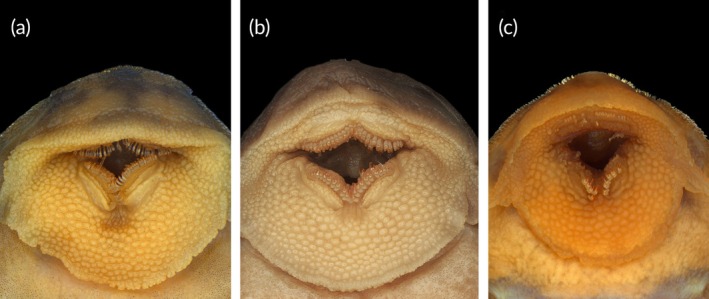
Comparison of the oral disc morphology in three morphologically similar armored catfish species: The teeth of *Peckoltia amjikin* (a) are bicuspid, with small and moderately narrow lateral cusps. *Peckoltia vittata* (b) has fewer teeth on both the premaxilla and dentary, with more robust and stout cusps. *Hypancistrus parkateje* (c) is distinguished by having an even lower number of teeth on both the premaxilla and dentary.

Maxillary barbels long, reaching past posterior edge of lower lip when extended posteriorly. Lips papillated. Teeth bicuspid, small and moderately narrow lateral cusp, mesial cusp larger and slender (Figure 4a). Dentary and premaxillary teeth differ in length; 15–27 teeth on dentary (20*, mode 19) and 14–26 on premaxilla (24*, mode 22). Dentaries angle 90°.

#### 3.1.6 Sexual dimorphism

None observed.

#### 3.1.7 Colouration

**FIGURE 5:**
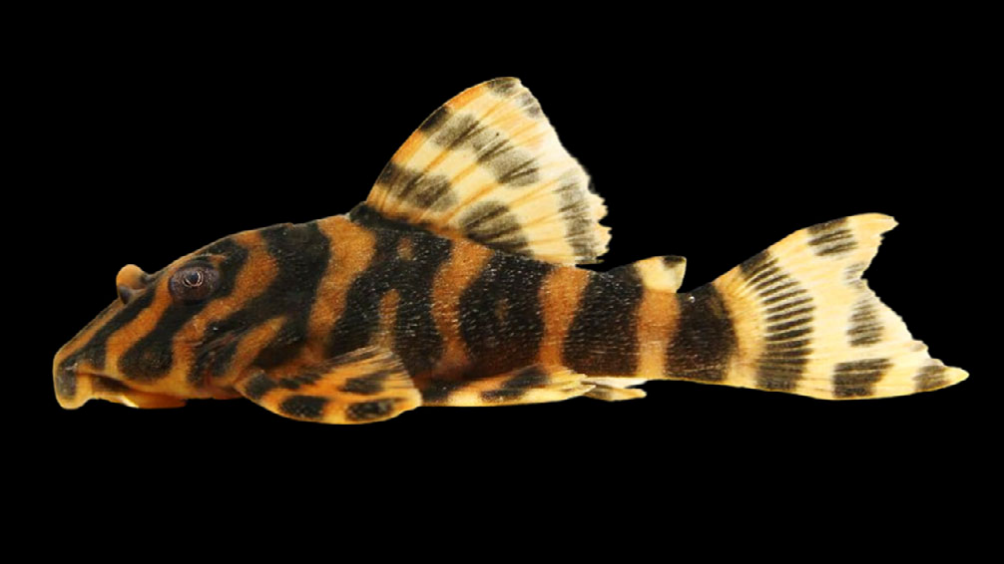
Live specimen of *Peckoltia amjikin*, collected in the rapids of the Rio Tocantins in São João do Araguaia city, PA, Brazil. Tan ground colouration is more evident than alcohol‐preserved specimens; the bars on snout, body and fin bars are more definite (Photo: Felipe Araújo).

Live specimens present tan‐yellowish ground colouration with four dark saddles from head to caudal peduncle. First body saddles ‘H’ shape. Saddle on dorsal, pectoral, adipose and caudal fins, in dorsal and caudal fins alternating from dark to light. In ethanol, body with dark banding pattern on light brown to pale yellow base. Three dark *E* oblique marks on tip of snout. A thin light grey bar on posterior of head, extending across branchial opening. Vertical vermicular bars from pectoral‐fin base to caudal peduncle; three dark bars in caudal fin. Ventral surface white to light grey with dark blotches on abdomen. Live specimens, with limits of body bars and markings on snout more prominent than described above (Figure 5).

### 3.2 Molecular phylogenetics

The mitochondrial Cytb sequences generated in this study are available on GenBank with accession number PV165471. The nuclear marker RAG1 was also sequenced for the new species but was not included in our phylogenetic analyses (PV165472).

**FIGURE 6:**
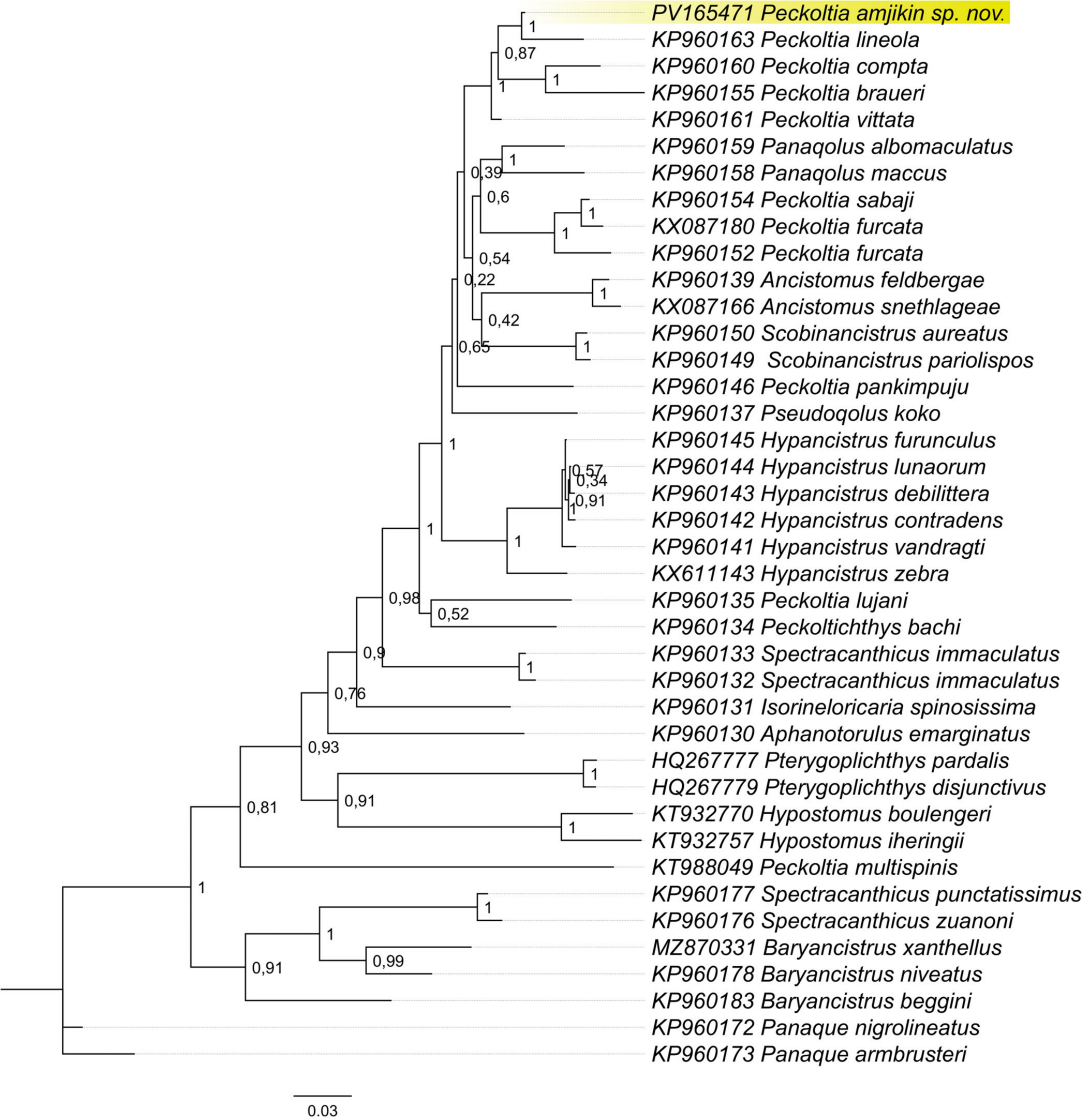
Phylogenetic reconstruction based on Cytb molecular markers of the major species of Loricariidae. The topology recovers the monophyly of *Peckoltia* although weak support values in the node branches. *Peckoltia amjikin* found positioned with congeners (yellow highlighted), confirming our morphological classification.

Both ML and BI phylogenetic reconstructions revealed low support values among species within the *Peckoltia* clade, with polytomous relationships observed across both methodological approaches, particularly at the nodes defining the Peckoltini tribe. Despite the overall low resolution, *P. amjikin* was consistently recovered within a well‐supported clade that also includes *Peckoltia lineola*, *P. compta*, *Peckoltia braueri* and *P. vittata* as the less‐derived species. For clarity, we chose to present only the BI topology in the main text (Figure 6). The ML tree can be found on Data S2.

#### 3.2.1 Geographical distribution

**FIGURE 7:**
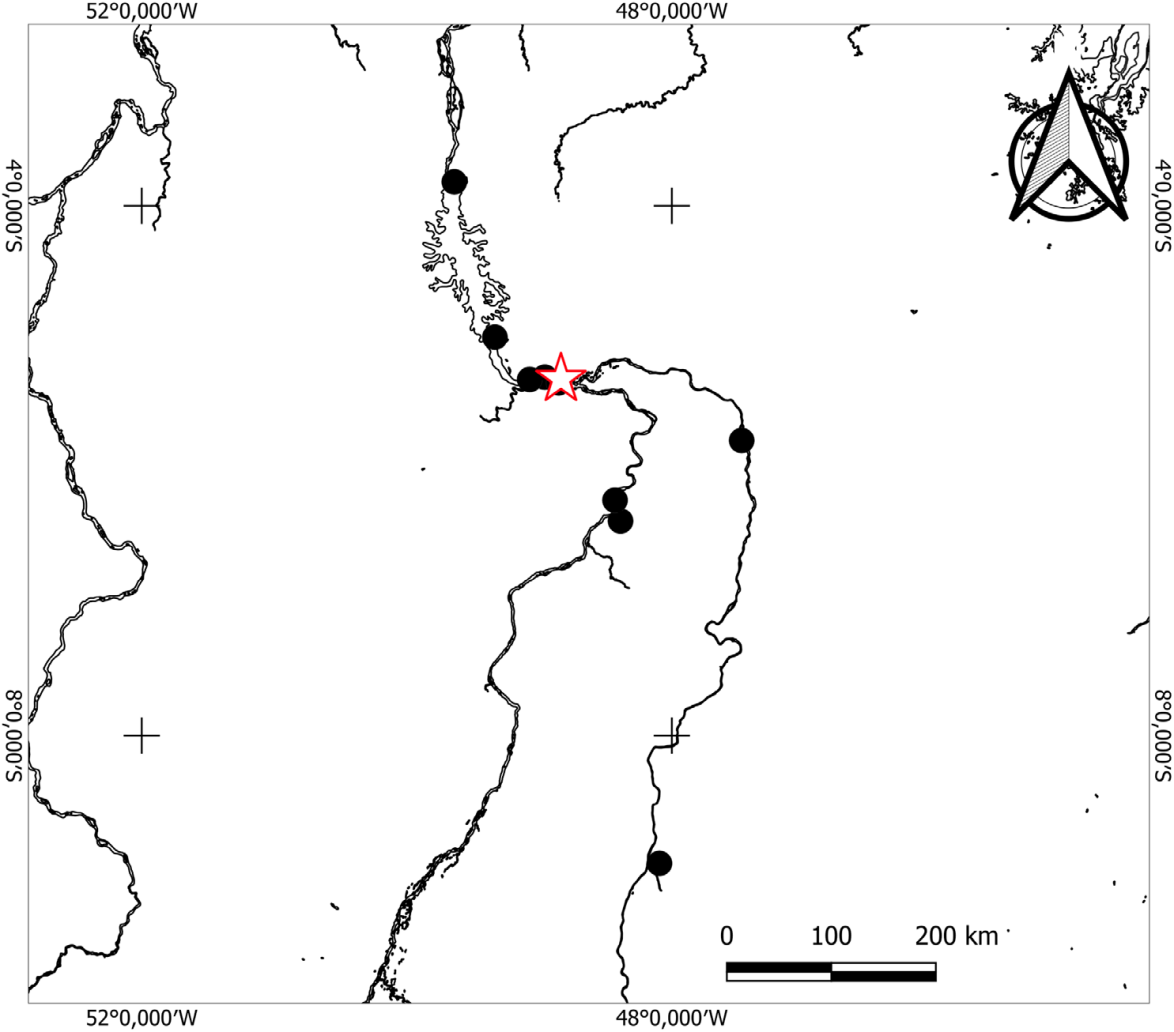
Distribution of *Peckoltia amjikin* throughout the Tocantins‐Araguaia River basin. Black circles stand for sampling sites; red star shows the type locality.

*Pecoltia amjikin* is found in bedrock background of moderate water flow along the Rio Tocantins‐Araguaia basin (Figure 7).

#### 3.2.2 Etymology

The specific epithet ‘amjikin’ is treated as a noun in opposition. ‘Amji kin’ refers to a state of happiness for the Krahô people, speakers of the Timbira language (Macro‐Jê trunk, Jês languages), and can be related to many cultural expressions within the Krahô's community, including singing, dancing, games, food bartering and ceremonial rituals. The Krahôs are located east of the Tocantins State in Brazil.

## 4 DISCUSSION

This paper describes a new species similar to the *P. vittata*, a morphotype to the rapids of Rio Tocantins‐Araguaia River basin. *Peckoltia amjikin* is distinguished from congeners by ventrally presenting diffuse blotches on ventral surface, body not presenting spots or blotches on the head, elongated odontodes on cheeks reaching the pectoral‐fin spine when relaxed against body, space between the eyes not entirely covered by a blotch, parieto‐supraoccipital moderately elevated not forming an apparent crest, by diminute plates with short odontodes on base of pectoral fins, on the pectoral girdle and in the anterior part of urogenital opening.

Considering internal anatomical comparisons, the new species differs from *P. vittata* by having a less‐developed suspensorium; absence of the paired slight grooves on the metapterygoid; a prominent lateral wall of the metapterygoid channel with a sturdy base and conspicuous, ornamented edges; the adductor palatine crest forming a minute, barely perceptible projection; and a small, concave, triangular‐shaped hyomandibula. Although our study presents this comparative approach, few works in the literature have addressed this anatomical bias, particularly due to the difficulty in accessing type material for detailed examination.

Major advance on *Peckoltia* species description in the past years had been resulted from revision of specimens deposited in museums, especially from morphotypes attributed as *P. vittata*. So, its wide, hypothetical distribution over the Amazon River basin suggests an assumption that its morphotypes may represent undescribed species (Armbruster, 2008), especially those that occur in the Brazilian territory. Like *P. compta*, *P. greedoi*, *P*. *lujani* and *P. wernekei*, these cases illustrate morphotypes to *P. vittata* that, with accurate taxonomic review, provided the description of new species (Armbruster & Lujan, 2016; Ribeiro et al., 2010). Therefore, careful attention is needed to precisely determine the *P. vittata* species group, avoiding identifications based on generic characters, such as the saddles on body, which are also found in congeneric species.

Our topology, with the inclusion of *P. amjikin*, recovered the monophyly of *Peckoltia*, as found in previous studies (Araújo et al., 2024; Lujan et al., 2015, 2017). Similarly, as reported in previous studies, the species clustering presents weak support values, with non‐evident separation among the lineages. This pattern was especially evident at nodes delimiting the Peckoltini tribe, where both ML and BI analyses showed polytomous clustering and low statistical support. Nevertheless, *P. amjikin* was consistently recovered within a well‐supported clade alongside *P. lineola*, *P. compta*, *P. braueri* and *P. vittata*, suggesting a closer evolutionary relationship among these taxa. These findings reinforce the hypothesis that diversification within *Peckoltia* may be relatively recent and possibly driven by Miocene hydrogeographic changes (~20–7 Ma) that promoted vicariance and dispersal across Amazonian freshwater systems, especially among loricariid armoured catfishes (Fišer et al., 2018) (Cassemiro et al., 2023; Dagosta & de Pinna, 2019; Fišer et al., 2018; Lujan et al., 2015).

Considering its distribution, *P. amjikin* occurs in the rapids of Rio Tocantins‐Araguaia basin, characterized by environment of intense hydrological dynamics (Lujan & Conway, 2015). In the Rio Araguaia, it has been recorded in the rapids of São Geraldo do Araguaia, Pará State (Middle Araguaia) and in the Rio Tocantins from the municipality of Peixe, Tocantins State (Upper Tocantins), to the north, to the rapids downstream of the Tucurui Hydroelectric Power Plant outflows. The species was first identified during the inventory of rapids in the Tocantins‐Araguaia basin by Araújo et al. (2025). It presents a colouration pattern similar to that of the recently described *Hypancistrus parkateje* (Araújo et al., 2024), a resemblance that may result from natural selection or even mimicry. Both species were frequently collected in sympatry at several sites, and distinguishing them required a close examination of specific morphological traits. The most reliable external diagnostic characters are found in the teeth cups: *H. parkateje* has long and wide teeth, with 5–8 dentary teeth (mode = 6) and 10–14 premaxillary teeth (mode = 12) (Figure 4c), whereas *P. amjikin* exhibits bicuspid teeth with a small, moderately narrow lateral cusp and a larger, slender mesial cusp (Figure 4a), with dentary and premaxillary teeth differing in length, 15–27 teeth on the dentary (mode = 19; holotype = 20) and 14–26 on the premaxilla (mode = 22; holotype = 24) (Araújo et al., 2024).

Mimetic interactions among Neotropical freshwater fishes have been documented in the literature among Loricariidae. For instance, *Otocinclus mimulus* mimics *Hoplisoma diphyes*, a chemically defended species from the Callichthyidae, forming mixed shoals in Paraguayan streams (Axenrot & Kullander, 2003). Similarly, *Otocinclus xakriaba*, from the São Francisco basin, resembles juveniles of *Brochis garbei*, suggesting a potential case of Batesian mimicry (Ribeiro & Pereira, 2002). These mimicry complexes highlight the role of visual convergence in predator avoidance and underscore the need for detailed morphological and ecological assessments when identifying sympatric species with similar colouration.

The Tocantins‐Araguaia basin has a diverse Hypostomini fauna, with at least 26 endemic species (Araújo et al., 2024, 2025; Chamon et al., 2022). This high endemism reflects a biogeographic mosaic typical of Amazonian drainages, especially in the plateau rivers of the Brazilian Shield, such as the Tocantins, Xingu and Tapajós, which favour isolation and speciation due to their rapids and elevated relief (Dagosta & de Pinna, 2017; Dagosta & de Pinna, 2019). However, further investigations into the biogeographic patterns of these species are necessary to improve the understanding of the evolution of rheophilic catfishes.

Therefore, this study is an advance in the taxonomy of one of the most diverse catfish groups of South America, describing a new species from a widely distributed morphospecies that represents putative undescribed species. Furthermore, despite the Tocantins‐Araguaia fish fauna has been well studied, the rapids habitats require more investigations to characterize its fish diversity, especially because it has been impacted by hydrological alteration by damming in the past four decades.

### 4.1 Comparative material examined

*Peckoltia brevis*: MPEG 38236, 1, 74.9 mm SL, Brazil, Pará, Rio Araguaia, Itupiranga, 4°57′40.68″ S 49°20′48.34″ W, 22 January 2018. MPEG 27422, 3, 79.2–96.9 mm SL, Brazil, Pará, Rio Tapajós, Jacareacanga, 5°3′32″ S 56°48′47″ W, 17 Mar. 2013. *Peckoltia oligospila*: MPEG 38435, 1, 105.0 mm SL, Brazil, Pará, Rio Araguaia, Itupiranga, 4°59′17.91″ S 49°19′53.27″ W, 22 January 2018. *P. vittata*: MPEG 13304, 2, 86.5–96.7 mm SL, Brazil, Rio Amazonas, Chaves, Pará, 0°10′22.4″ S 49°56′53″ W, 13 January 2007. MPEG 13322, 1, 90.4 mm SL, Brazil, Rio Amazonas, Chaves, Pará, 0°10′22.4″ S 49°56′53″ W, 13 January 2007. MCZ 7999, 1, photography, Brazil, Pará, Rio Xingu, Melgaço, Furo do Tajapuru, 1°50′30.0″ S 50°25′30.0″ W, 2 August 1865. MPEG 13428, 1, 100.9 mm SL, Brazil, Rio Xingu, Altamira, Pará, 3°12′48″ S 52°12′41.7″ W, 1 October 2002. MPEG 13428, 1, 100.3 mm SL, Brazil, Rio Xingu, Altamira, Pará, 3°12′48″ S 52°12′41.7″ W, 1 October 2002. MPEG 21634, 1, 82.3 mm SL, Brazil, Pará, Rio Xingu, Senador José Porfírio, 3°35′49.11″ S 51°54′2.65″ W, 8 March 2011. *Peckoltia sabaji*: LIA 007487, 1, 122.9 mm SL, Brazil, Rio Xingu, Altamira, Pará, 2°53′18.8″ S 51°56′26.0″ W, 21 October 2020. MPEG 039440, 1, 127.9 mm SL, Brazil, Rio Xingu, Altamira, Pará, 3°17′58.5″ S 51°42′29.7″ W, 27 February 2021. MPEG 039441, 1, 121 mm SL, Brazil, Rio Xingu, Altamira, Pará, 3°15′38.9″ S 51°40′24.0″ W, 1 February 2021.

## AUTHOR CONTRIBUTIONS

Felipe Arian Andrade de Araújo: conceptualization, method, manuscript writing and editing. Marlon Felipe Chumber Ferreira: methodology, manuscript writing and editing. Aline Nascimento Silva: data collection, manuscript writing and editing. Wolmar Benjamin Wosiacki: funding, manuscript writing and editing, critic review.

## FUNDING INFORMATION

This research was supported by financial contributions from the Fundação Coordenação de Aperfeiçoamento de Pessoal de Nível Superior (CAPES), Fundo de Defesa de Direito Difusos (FDD), Financiadora de Estudos e Projetos (FINEP) and Conselho Nacional de Desenvolvimento Científico e Tecnológico (CNPq). The Article Processing Charge for the publication of this research was funded by the Coordenação de Aperfeiçoamento de Pessoal de Nível Superior‐Brasil (CAPES) (ROR identifier: 00x0ma614).

## Supporting information


**Data S1.** Complete morphometric dataset of type specimens.

## References

[jfb70235-bib-0001] Araújo, F. , Ferreira, M. , Monteiro, I. , & Wosiacki, W. (2024). A new species of *Hypancistrus* Isbrücker & Nijssen 1991 (Loricariidae, Siluriformes) from the rapids of the middle Rio Tocantins. Journal of Fish Biology, 106, 592–601. 10.1111/jfb.15971 39505832

[jfb70235-bib-0002] Araújo, F. , Monteiro, I. , Jacob, L. L. , Leão, R. , Duarte, A. , Leonardo, M. , Nascimento, J. , Ferreira, M. , Mendonça, M. , Wosiacki, W. , Sousa, L. , & Akama, A. (2025). An exploratory survey of fish species inhabiting rapids from the Tocantins‐Araguaia River basin: Perspectives on species diversity and conservation. Biota Neotropica, 25, 1–16. 10.1590/1676-0611

[jfb70235-bib-0004] Armbruster, J. W. (2003). *Peckoltia sabaji*, a new species from the Guyana shield (Siluriformes: Loricariidae). Zootaxa, 344, 1. 10.11646/zootaxa.344.1.1

[jfb70235-bib-0005] Armbruster, J. W. (2008). The genus *Peckoltia* with the description of two new species and a reanalysis of the phylogeny of the genera of the Hypostominae (Siluriformes: Loricariidae). Zootaxa, 1822, 1–76. 10.11646/zootaxa.1822.1.1

[jfb70235-bib-0006] Armbruster, J. W. , & Lujan, N. K. (2016). A new species of *Peckoltia* from the upper Orinoco (Siluriformes, loricariidae). ZooKeys, 2016, 105–121. 10.3897/zookeys.569.6630 PMC4829682 27110153

[jfb70235-bib-0007] Armbruster, J. W. , & Lujan, N. K. (2024). New tribe‐level classification of Hypostominae (Loricariidae) based on optimization of morphological states on DNA‐based relationships, with descriptions of three new tribes and two new genera. Neotropical Ichthyology, 22, e240108. 10.1590/1982-0224-2024-0108

[jfb70235-bib-0003] Armbruster, J. , van der Sleen, P. , & Lujan, N. (2018). Family Loricariidae ‐ suckermouth armored catfishes. In P. van der Sleen & J. Albert (Eds.), Field guide to the fishes of the Amazon, Orinoco, and Guianas (pp. 253–286). Princeton University press.

[jfb70235-bib-0008] Armbruster, J. W. , & Werneke, D. C. (2005). *Peckoltia cavatica*, a new loricariid catfish from Guyana and a redescription of *P. Braueri* (Eigenmann 1912) (Siluriformes). Zootaxa, 882, 1–14.

[jfb70235-bib-0009] Armbruster, J. W. , Werneke, D. C. , & Tan, M. (2015). Three new species of saddled loricariid catfishes, and a review of *Hemiancistrus*, *Peckoltia*, and allied genera (Siluriformes). ZooKeys, 480, 97–123. 10.3897/zookeys.480.6540 PMC4319111 25685026

[jfb70235-bib-0010] Axenrot, T. , & Kullander, S. (2003). *Corydoras diphyes* (Siluriformes: Callichthyidae) and *Otocinclus mimulus* (Siluriformes: Loricariidae), two new species of catfishes from Paraguay, a case of mimetic association. Ichthyological Explorantion of Freshwaters, 14, 249–272.

[jfb70235-bib-0011] Cassemiro, F. A. S. , Albert, J. S. , Antonelli, A. , Menegotto, A. , Wüest, R. O. , Cerezer, F. , Coelho, M. T. P. , Reis, R. E. , Tan, M. , Tagliacollo, V. , Bailly, D. , da Silva, V. F. B. , Frota, A. , da Graça, W. J. , Ré, R. , Ramos, T. , Oliveira, A. G. , Dias, M. S. , Colwell, R. K. , … Graham, C. H. (2023). Landscape dynamics and diversification of the megadiverse south American freshwater fish fauna. Proceedings of the National Academy of Sciences of the United States of America, 120, e2211974120. 10.1073/pnas.2211974120 36595684 PMC9926176

[jfb70235-bib-0028] Chamon, C. , Serra, J. , Camelier, P. , Zanata, A. , Fichberg, I. , & Marinho, M. (2022). Building knowledge to save species: 20 years of ichthyological studies in the Tocantins‐Araguaia River basin. Biota Neotropica, 22(2), e20211296. 10.1590/1676-0611-BN-2021-1296

[jfb70235-bib-0012] Dagosta, F. C. P. , & de Pinna, M. (2017). Biogeography of amazonian fishes: Deconstructing river basins as biogeographic units. Neotropical Ichthyology, 15, e170034. 10.1590/1982-0224-20170034

[jfb70235-bib-0013] Dagosta, F. C. P. , & de Pinna, M. (2019). The fishes of the Amazon: Distribution and biogeographical patterns, with a comprehensive list of species. Bulletin of the American Museum of Natural History, 2019, 1–63. 10.1206/0003-0090.431.1.1

[jfb70235-bib-0014] Edler, D. , Klein, J. , Antonelli, A. , & Silvestro, D. (2021). raxmlGUI 2.0: A graphical interface and toolkit for phylogenetic analyses using RAxML. Methods in Ecology and Evolution, 12, 373–377. 10.1111/2041-210X.13512

[jfb70235-bib-0015] Fedorov, A. , Beichel, R. , Kalpathy‐Cramer, J. , Finet, J. , Fillion‐Robin, J.‐C. , Pujol, S. , Bauer, C. , Jennings, D. , Fennessy, F. , Sonka, M. , Buatti, J. , Aylward, S. , Miller, J. V. , Pieper, S. , & Kikinis, R. (2012). 3D slicer as an image computing platform for the quantitative imaging network. Magnetic Resonance Imaging, 30, 1323–1341. 10.1016/j.mri.2012.05.001 22770690 PMC3466397

[jfb70235-bib-0016] Fišer, C. , Robinson, C. T. , & Malard, F. (2018). Cryptic species as a window into the paradigm shift of the species concept. Molecular Ecology, 27, 613–635. 10.1111/mec.14486 29334414

[jfb70235-bib-0017] Fricke, R. , Eschmeyer, W. , & Van der Laan, R. (2025). Eschmeyer's catalog of fishes: genera, species, references. https://researcharchive.calacademy.org/research/ichthyology/catalog/fishcatmain.asp

[jfb70235-bib-0018] Lanfear, R. , Frandsen, P. B. , Wright, A. M. , Senfeld, T. , & Calcott, B. (2016). PartitionFinder 2: New methods for selecting partitioned models of evolution for molecular and morphological phylogenetic analyses. Molecular Biology and Evolution, 34, 772–773. 10.1093/molbev/msw260 28013191

[jfb70235-bib-0020] Lujan, N. K. , Armbruster, J. W. , Lovejoy, N. R. , & López‐Fernández, H. (2015). Multilocus molecular phylogeny of the suckermouth armored catfishes (Siluriformes: Loricariidae) with a focus on subfamily Hypostominae. Molecular Phylogenetics and Evolution, 82, 269–288. 10.1016/j.ympev.2014.08.020 25193609

[jfb70235-bib-0019] Lujan, N. , & Conway, K. (2015). Life in the fast line: A review of rheophily in freshwater fishes. In R. Riesch , M. Tobler , & M. Plath (Eds.), Extremophile fishes: Ecology, evolution and physiology of teleost in extreme environments (pp. 107–136). Springer International Publishing.

[jfb70235-bib-0021] Lujan, N. K. , Cramer, C. A. , Covain, R. , Fisch‐Muller, S. , & López‐Fernández, H. (2017). Multilocus molecular phylogeny of the ornamental wood‐eating catfishes (Siluriformes, Loricariidae, *Panaqolus* and *Panaque*) reveals undescribed diversity and parapatric clades. Molecular Phylogenetics and Evolution, 109, 321–336. 10.1016/j.ympev.2016.12.040 28065866

[jfb70235-bib-0023] Ribeiro, R. , Oliveira, D. E. , Zuanon, J. , Rapp Py‐Daniel, L. , & Rocha, M. S. (2010). *Peckoltia compta*, a new species of catfish from the Brazilian Amazon, rio Tapajós basin (Siluriformes: Loricariidae). Zootaxa, 2534, 48–56.

[jfb70235-bib-0022] Ribeiro, A. C. , & Pereira, E. H. L. (2002). A new species of *Parotocinclus* (Siluriformes: Loricariidae) from the rio São Francisco basin, southeastern Brazil. Ichthyological Exploration of Freshwaters, 13, 231–238.

[jfb70235-bib-0024] Ronquist, F. , Teslenko, M. , Van Der Mark, P. , Ayres, D. L. , Darling, A. , Höhna, S. , Larget, B. , Liu, L. , Suchard, M. A. , & Huelsenbeck, J. P. (2012). Mrbayes 3.2: Efficient bayesian phylogenetic inference and model choice across a large model space. Systematic Biology, 61, 539–542. 10.1093/sysbio/sys029 22357727 PMC3329765

[jfb70235-bib-0025] Sabaj, M. H. (2020). Codes for natural history collections in ichthyology and herpetology. Copeia, 108, 593–669. 10.1643/ASIHCODONS2020

