# Supplementary material for: A new species of Peckoltia (Siluriformes, Loricariidae) from the rapids of the Rio Tocantins‐Araguaia basin, Brazil
Source: J Fish Biol. 2025 Oct 4;108(1):351–60. doi: 10.1111/jfb.70235 (PMC13033947; doi:10.1111/jfb.70235)
Supplement: Supplementary file 1 — Data S1. Complete morphometric dataset of type specimens. [file JFB-108-351-s001.zip › Raxml tree.pdf]

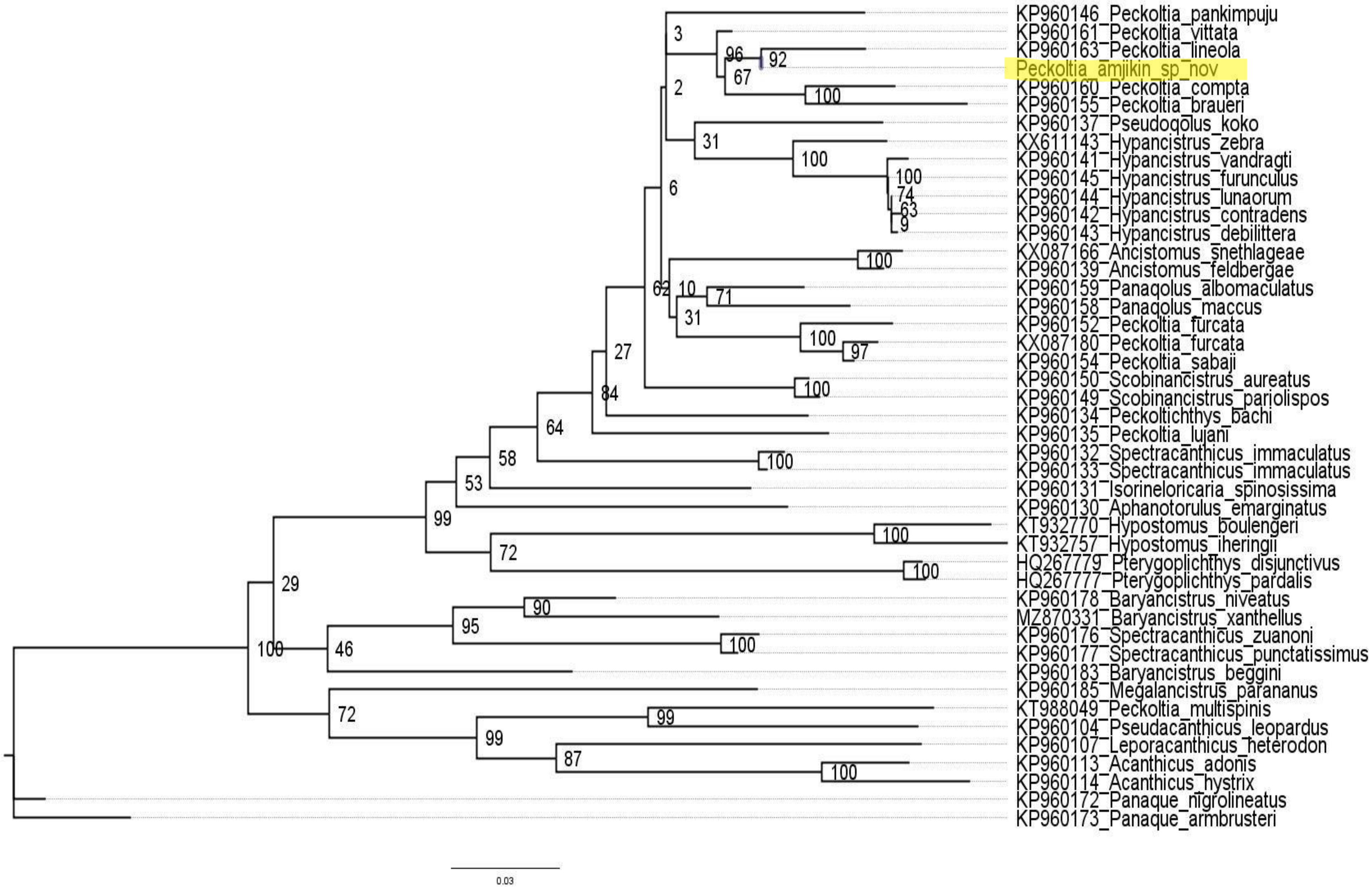

Figure S1. Maximum Likelihood (ML) phylogenetic tree based on the mitochondrial Cytb gene, supporting the placement of *Peckoltia amjikin* (highlighted in yellow) within the genus *Peckoltia*.

[illegible]
